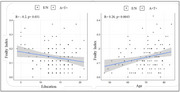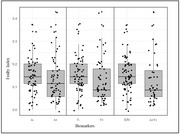# Unraveling the link between frailty and Alzheimer's disease biomarkers in patients with mild cognitive impairment

**DOI:** 10.1002/alz70856_098801

**Published:** 2025-12-24

**Authors:** Simona Buscarnera, Marco Canevelli, Giuseppe Bruno, Valentina Garibotto, Giovanni B Frisoni, Federica Ribaldi

**Affiliations:** ^1^ Sapienza University of Rome, Rome, Italy; ^2^ Sapienza University of Rome, Rome, Rome, Italy; ^3^ Department of Human Neurosciences, Sapienza University of Rome, Roma, Italy; ^4^ Centre for Biomedical Imaging (CIBM), Geneva, Switzerland; ^5^ Division of Nuclear Medicine and Molecular Imaging, Geneva University Hospitals, Geneva, Switzerland; ^6^ Laboratory of Neuroimaging and Innovative Molecular Tracers (NIMTlab), Geneva University Neurocenter and Faculty of Medicine, University of Geneva, Geneva, Switzerland; ^7^ Laboratory of Neuroimaging of Aging (LANVIE), University of Geneva, Geneva, Switzerland; ^8^ Geneva Memory Center, Department of Rehabilitation and Geriatrics, Geneva University Hospitals, Geneva, Switzerland; ^9^ Geneva Memory Center, Department of Rehabilitation and Geriatrics, Geneva University Hospitals, Geneva, Geneva, Switzerland

## Abstract

**Background:**

Alzheimer's disease (AD) can be identified through biomarkers of amyloid (A) and tau (T) pathology. Frailty, a measure of biological aging, could impact the association between AD neuropathology and its clinical manifestation. We aimed to investigate the relationship between frailty and AD biomarkers among people with mild cognitive impairment (MCI) attending a university memory clinic.

**Method:**

Data were collected from a cohort of patients with MCI at the Memory Center of Geneva University Hospital (Switzerland). Frailty was assessed using a 35‐item frailty index (FI). A and T positivity were determined through amyloid and tau PET or CSF analysis. Participants were divided into two subgroups: i) A+T+ (both amyloid and tau positive), and ii) E/N (either A+ or T+, neither A+ nor T+), including all other combinations of A/T status. We first explored the correlation between FI, age, and education. Demographics, FI scores, and neuropsychological test results were then compared between these two groups. Logistic regression models, adjusted for age, sex, and education, were used to examine the association between FI and AT positivity.

**Result:**

120 patients were included. FI was positively correlated with age and inversely with education. A+T+ patients exhibited lower FI scores compared to E/N participants (0.13 ± 0.10 vs. 0.15 ± 0.08, *p* = 0.01). Logistic regressions found a negative association between FI and A+T+ (OR 0.6, 95% CI 0.32 – 0.90; *p* = 0.02).

**Conclusion:**

Frailty is associated with a lower likelihood of AD biomarker positivity in patients with MCI. Frailty might reflect alternative pathophysiological mechanisms contributing to cognitive impairment.